# Heterodimer-heterotetramer formation mediates enhanced sensor activity in a biophysical model for BMP signaling

**DOI:** 10.1371/journal.pcbi.1009422

**Published:** 2021-09-30

**Authors:** Md. Shahriar Karim, Aasakiran Madamanchi, James A. Dutko, Mary C. Mullins, David M. Umulis

**Affiliations:** 1 Agricultural and Biological Engineering, Purdue University, West Lafayette, Indiana, United States of America; 2 Department of Electrical and Computer Engineering, North South University, Dhaka, Bangladesh; 3 Weldon School of Biomedical Engineering, Purdue University, West Lafayette, Indiana, United States of America; 4 Polytechnic Institute, Purdue University, West Lafayette, Indiana, United States of America; 5 University of Pennsylvania Perelman School of Medicine, Philadelphia, Pennsylvania, United States of America; University of Pittsburgh, UNITED STATES

## Abstract

Numerous stages of organismal development rely on the cellular interpretation of gradients of secreted morphogens including members of the Bone Morphogenetic Protein (BMP) family through transmembrane receptors. Early gradients of BMPs drive dorsal/ventral patterning throughout the animal kingdom in both vertebrates and invertebrates. Growing evidence in Drosophila, zebrafish, murine and other systems suggests that BMP ligand heterodimers are the primary BMP signaling ligand, even in systems in which mixtures of BMP homodimers and heterodimers are present. Signaling by heterodimers occurs through a hetero-tetrameric receptor complex comprising of two distinct type one BMP receptors and two type II receptors. To understand the system dynamics and determine whether kinetic assembly of heterodimer-heterotetramer BMP complexes is favored, as compared to other plausible BMP ligand-receptor configurations, we developed a kinetic model for BMP tetramer formation based on current measurements for binding rates and affinities. We find that contrary to a common hypothesis, heterodimer-heterotetramer formation is *not* kinetically favored over the formation of homodimer-tetramer complexes under physiological conditions of receptor and ligand concentrations and therefore other mechanisms, potentially including differential kinase activities of the formed heterotetramer complexes, must be the cause of heterodimer-heterotetramer signaling primacy. Further, although BMP complex assembly favors homodimer and homomeric complex formation over a wide range of parameters, ignoring these signals and instead relying on the heterodimer improves the range of morphogen interpretation in a broad set of conditions, suggesting a performance advantage for heterodimer signaling in patterning multiple cell types in a gradient.

## Author summary

TGF-β signaling is an important cell signaling system through which cells respond to external information. In the TGF-β system, signaling is initiated when a ligand dimer pair binds to a receptor tetramer. Interestingly, in numerous developmental contexts, TGF-β signaling has a greater response to heterodimeric ligands (dimers of multiple ligands), as compared to homomeric ligands (dimers made of two molecules of a single ligand). However, neither the cause of heterodimer signaling primacy, nor the systemic effects of heterodimeric vs homomeric signaling are understood. We use a biophysically-informed computational modeling approach to investigate the system dynamics of heterodimer-heterotetramer BMP signaling, to understand the cause and consequence of the requirement for Bmp2/7-mediated signaling in dorsoventral patterning in zebrafish development. Using our model, we demonstrate that BMP heterodimer signaling complex formation is not kinetically favored over homodimer signaling complexes, suggesting subfunctionalization of BMP receptors may be required to explain heterodimer signaling. Additionally, we show that heterodimer signaling provides a performance advantage via increased range of morphogen interpretation. Our findings provide insight into the systems principles involved in developmental signaling.

## Introduction

**Fig 1:**
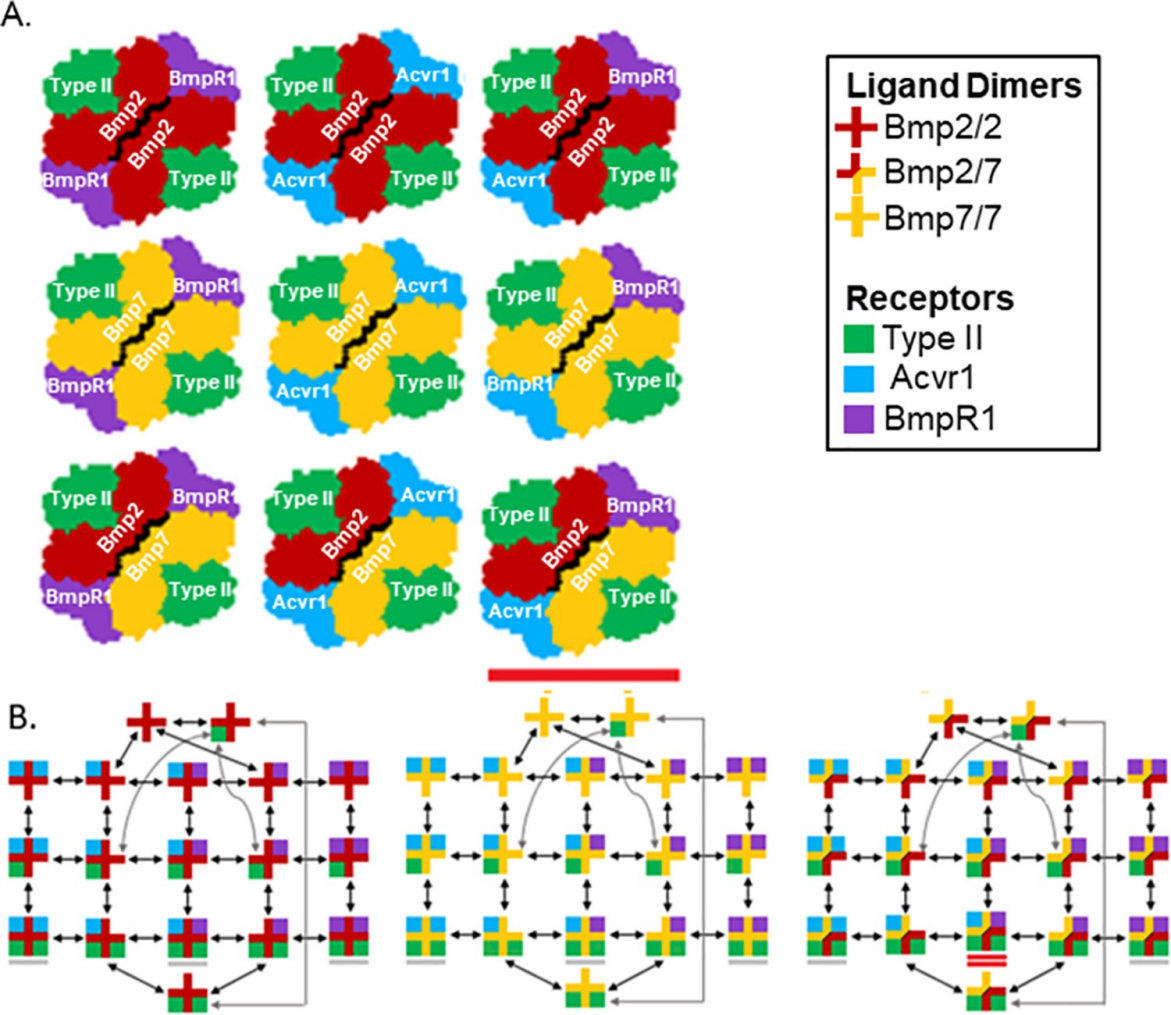
BMP ligand receptor interactions and oligomerization network. **A.** Cartoon depiction of ‘overhead view’ of BMP ligand-dimer receptor-tetramer complexes. BMP signaling occurs through an oligomeric signaling complex which necessarily consists of a ligand dimer (red/gold plus) bound to a receptor tetramer. The receptor tetramer requires two Type II receptors (green square) and two Type I receptors (BmpR1 in purple and/or Acvr1 in cyan). The heterodimer-heterotetramer is underlined in red. **B.** Network of BMP ligand-receptor interactions. Oligomeric signaling complexes are formed through reversible reactions (double-sided arrows). Tetrameric complexes are underlined in the figure above. The heterodimer-heterotetramer, Bmp2/7-Acvr1-BmpR1-(Type II)_2_ is double underlined.

The differentiation of biological systems is directed by the interpretation of biochemical morphogen gradients in a concentration-dependent manner. The remarkable robustness and precision of developmental patterning reveals careful organization of the underlying developmental signaling systems. These systems are highly evolutionarily conserved, and through the use of mathematical modeling, the foundational principles that guide the evolution of these systems are beginning to be understood. The Bone Morphogenetic Protein (BMP) signaling pathway is an essential signaling system that regulates development as well as a wide range of cellular processes in both vertebrates and invertebrates [1–4]. In canonical BMP signaling, secreted ligands form dimeric complexes, both homodimers and heterodimers, which bind to membrane-bound BMP receptors belonging to the receptor serine-threonine kinase family. Upon ligand binding, BMP receptors oligomerize, as depicted in Fig 1A and 1B, into tetrameric signaling complexes that consist of two type I and two type II kinase receptors [1,2]. Ligand-bound tetrameric signaling complexes initiate an intracellular signaling cascade by instigating the phosphorylation of BMP-responsive Smad proteins [2,5,6]. Finally, the phosphorylated Smad (pSmad) forms complexes with co-Smad and accumulates in the nucleus to regulate gene expression [6].

Interestingly, in several biological systems BMP ligand heterodimers demonstrate stronger biological activity than BMP ligand homodimers. In *Drosophila* development, BMP ligand homologues Decapentaplegic (Dpp) and Screw (Scw) signal through two type I receptors (Saxophone and Thickvein) and two type II receptors (Punt) [2]. Dpp-Scw heterodimers induce downstream signaling at approximately 10-fold the rate of equimolar Dpp homodimer, and heterodimer signaling is more robust in response to changes in gene dosage [2]. Heterodimer signaling also plays critical roles in *Drosophila* wing disc and wing vein patterning [7,8].

The primacy of BMP heterodimer signaling is conserved evolutionarily and has been identified in several vertebrate contexts including, bone regeneration, axon guidance, and stem cell differentiation [1,2,9–12]. In the vertebrate early embryo the significance of BMP heterodimer signaling is more exaggerated; in zebrafish, BMP-induced pattering is *exclusively* mediated by heterodimers [1,13]. Mutant experiments reveal that loss of either *bmp2b* or *bmp7a* is sufficient to induce a complete loss of BMP signaling in the embryo [13]. Further, recombinant Bmp2 and Bmp7 homodimers are unable to signal in the zebrafish and only recombinant Bmp2/7 heterodimer is able to rescue BMP signaling [1]. Additionally, BMP signaling in embryonic development is mediated through a specific heterotetrameric BMP receptor complex. Specifically, two distinct BMP Type I receptors, BmpRI (Alk3/6), and Acvr1 (Alk2/8) are required for BMP-mediated dorsoventral patterning in zebrafish.

Despite the experimental evidence for the centrality of BMP heterodimer-heterotetramer signaling during embryogenesis, the system properties that privilege heterodimer-heterotetramer signaling in the BMP system remain unclear [1,2,14]. Previously it has been suggested that the heterodimer-heterotetramer may simply be the predominant form of BMP ligand-receptor complex; a consequence of enhanced heterotetramer formation due to the higher combined affinity for the type I and type II receptors by the heterodimer ligand [1]. To test this hypothesis, and to characterize the systems level properties of BMP ligand-induced receptor oligomerization, we developed a physiologically-based mathematical model of ligand-receptor complex formation based on the receptor binding affinities of BMP ligands and the kinetics of subsequent receptor oligomerization. Data from our computational model indicates that privileged heterodimer-heterotetramer signaling is likely not mediated by favored assembly of that specific oligomeric signaling complex. In fact, no combination of receptor and ligand concentrations produces conditions in which heterodimer-heterotetramer complexes make up a majority of tetrameric ligand-receptor complexes, and it is only under very stringent conditions that heterodimer-heterotramer complexes constitute a plurality of tetrameric complexes. The absence of conditions supporting predominant formation of heterodimer-heterotetramer complexes suggests that alternative hypotheses such as differential kinase activity or a role for specific inhibition by secreted antagonists or co-receptors are needed to explain dominance [15].

Intriguingly, however, BMP heterodimer-heterotetramer signaling in the simulations exhibit important performance advantages over other ligand receptor complexes- suggesting a selective advantage for signaling via heterodimer-heterotetramer in pattern formation or cell signaling control. Specifically, the heterodimer-heterotetramer exhibits a larger dynamic range than all other plausible BMP ligand dimer-receptor tetramer complexes in a broad set of parameter conditions. In other words, the BMP heterodimer-heterotetramer acts as a superior sensor compared to other BMP ligand-receptor combinations because its ligand input concentrations correspond linearly to tetrameric receptor signaling complex levels over a larger concentration range. Recent computational modeling work shows [16,17] that in vertebrate systems, the BMP morphogen gradient is carefully regulated and therefore our finding of enhanced sensor function is a viable mechanism for the experimentally observed phenomenon of heterodimer dominance in BMP signaling.

## Results

A natural hypothesis to explain heterodimer dominance, i.e the requirement for BmpR1-Acvr1-(Type II)_2_ heterotetramer signaling, is that the kinetics of Bmp2/7 ligand binding to receptors favors the formation of this species over the other eight tetrameric signaling complexes (Fig 1B). To screen the network, we simulated the model 84,375 times (Table 1) to steady-state with parameters for ligand and receptor concentrations over the physiologically expected range in vivo (0.01 to 1 nM ligand; 1 to 50 nM receptor, corresponding to ~360 to 18,000 receptors per cell) [5,18–20]. Additionally, we screened reduction of dimensionality adjustments (Surface Enhancement Factor γ, values of 10, 50, 100, 500, and 1000). Values for γ between 10 and 100 are physiologically reasonable (see S2 Text). Values of 500 and 1000 are included to test the robustness of our solutions against these assumptions. (Table 1).

**Table 1:** 84,375 Point Parameter Screen.

Parameter	Values	Grid Points	Comments
**Bmp ligand**	1 × [10^−2^ to 10^0^] nM	5	Bmp2/2 = Bmp7/7 = Bmp2/7
**BmpR1**	[1 to 50] nM	15	
**Acvr1**	[1 to 50] nM	15	
**Type II**	[1 to 50] nM	15	
**γ**	[10, 50, 100, 500, 1000]	5	Surface Enhancement Factor (SEF)

**Fig 2:**
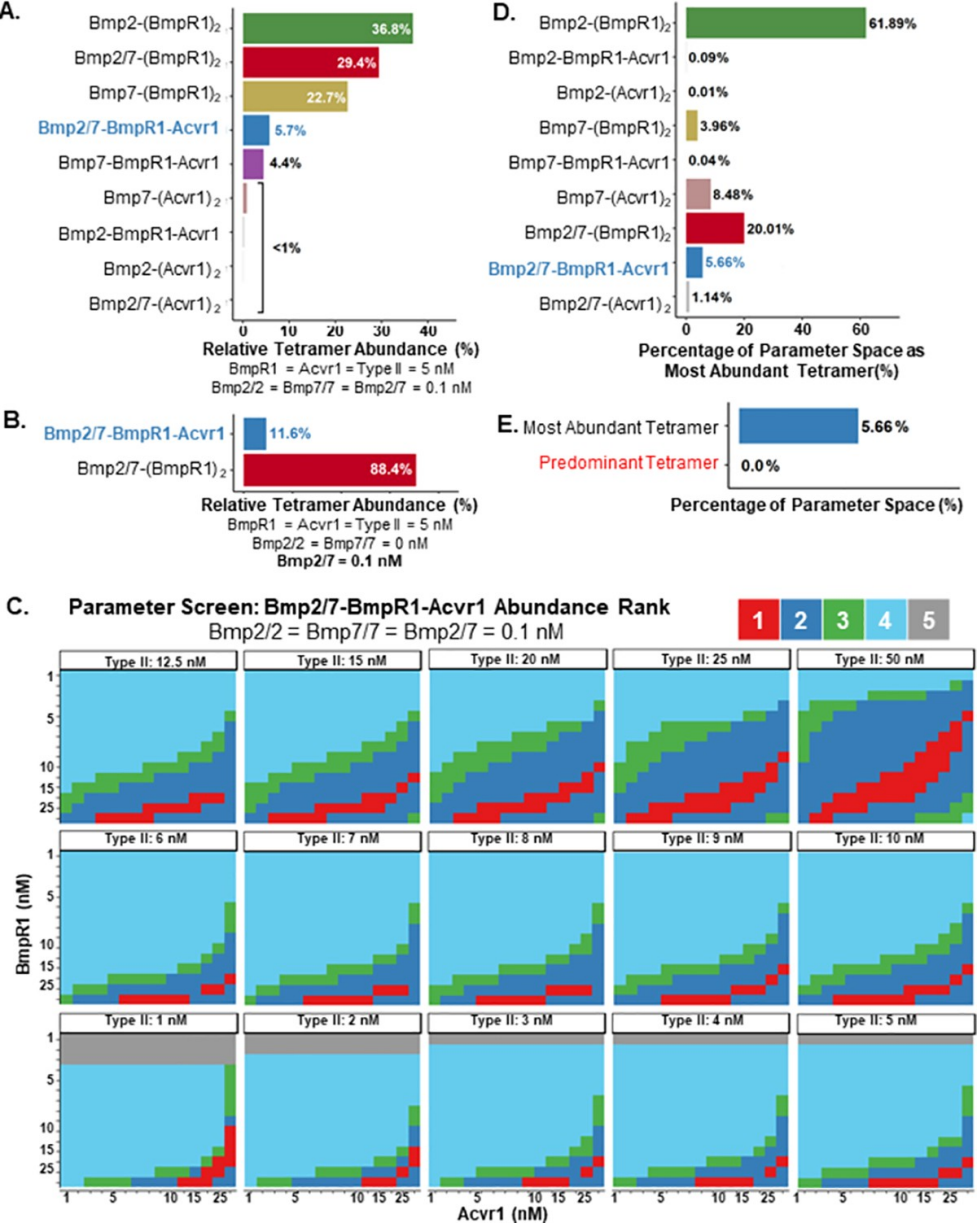
Oligomerization kinetics do not explain heterodimer-heterotetramer dominance. **A.** Proportion of ligand-receptor tetrameric complexes at equimolar receptor at physiological conditions. The heterodimer-heterotetramer is only the fourth most abundant tetrameric complex. **B.** Proportion of ligand-receptor tetrameric complexes in a simulation with no homodimer ligand. Heterodimer-heterotetramer production is still not kinetically favored. **C.** Visualization of the prevalence of heterodimer-heterotetramer compared to other ligand-receptor tetramers in 3,375 points of a 84,375 point screen. The red region represents parameter space in which the heterodimer-heterotetramer is the most prevalent species (3.84% of this sample. **D.** The portion of the parameter space in which each ligand-receptor tetrameric complex is the most abundant. **E.** The portion of the parameter space in which the heterodimer-heterotetramer is the most abundant ligand-receptor tetrameric complex (5.66%) and the predominant tetrameric complex (0.0%).

As shown in Fig 2A, under physiologically relevant ligand and receptor concentrations, the most abundant receptor-tetramer complexes contain two BmpR1 receptors: Bmp2-(BmpR1)_2_-(Type II)_2_ is the most prevalent, followed by Bmp2/7-(BmpR1)_2_-(Type II)_2_, and then Bmp7-(BmpR1)_2_-(Type II)_2_. The fourth most-abundant complex is Bmp2/7-Acvr1-BmpR1-(Type II)_2_, the required signaling complex in embryonic dorsoventral patterning. Even when the homodimer ligands are removed from the model, leaving only the Bmp2/7 ligand, ligand and receptors favor formation of the Bmp2/7 complex with two BmpR1 and two type II receptors (Fig 2B). The heterodimer-heterotetramer signaling complex, Bmp2/7-Acvr1-BmpR1-(Type II)_2_, was the most abundant tetrameric signaling complex in only 5.66% of the tested parameters (Fig 2D and 2E). In contrast, Bmp2-(BmpR1)_2_-(Type II)_2_ and Bmp2/7-(BmpR1)_2_-(Type II)_2_ were the most abundant tetramer complexes in 61.89% and 20.01% of parameter conditions (Fig 2D). Surprisingly, we did not find a single solution in which the heterodimer-heterotetramer complex formation was predominant, as defined by a greater concentration than the total of the homodimer receptor complexes (Fig 2E). A visualization of a subset of the parameter space is shown in Fig 2C. The red region represents the 3.84% of physiologically relevant parameter space in which the heterodimer-heterotetramer is the most abundant tetramer. Notably, limited BmpR1 availability compared to the other receptors is a hallmark of this portion of the parameter space (S1 and S2 Figs).

### Conditions and behavior of the systems under alternative hypotheses of heterodimer dominance

**Fig 3:**
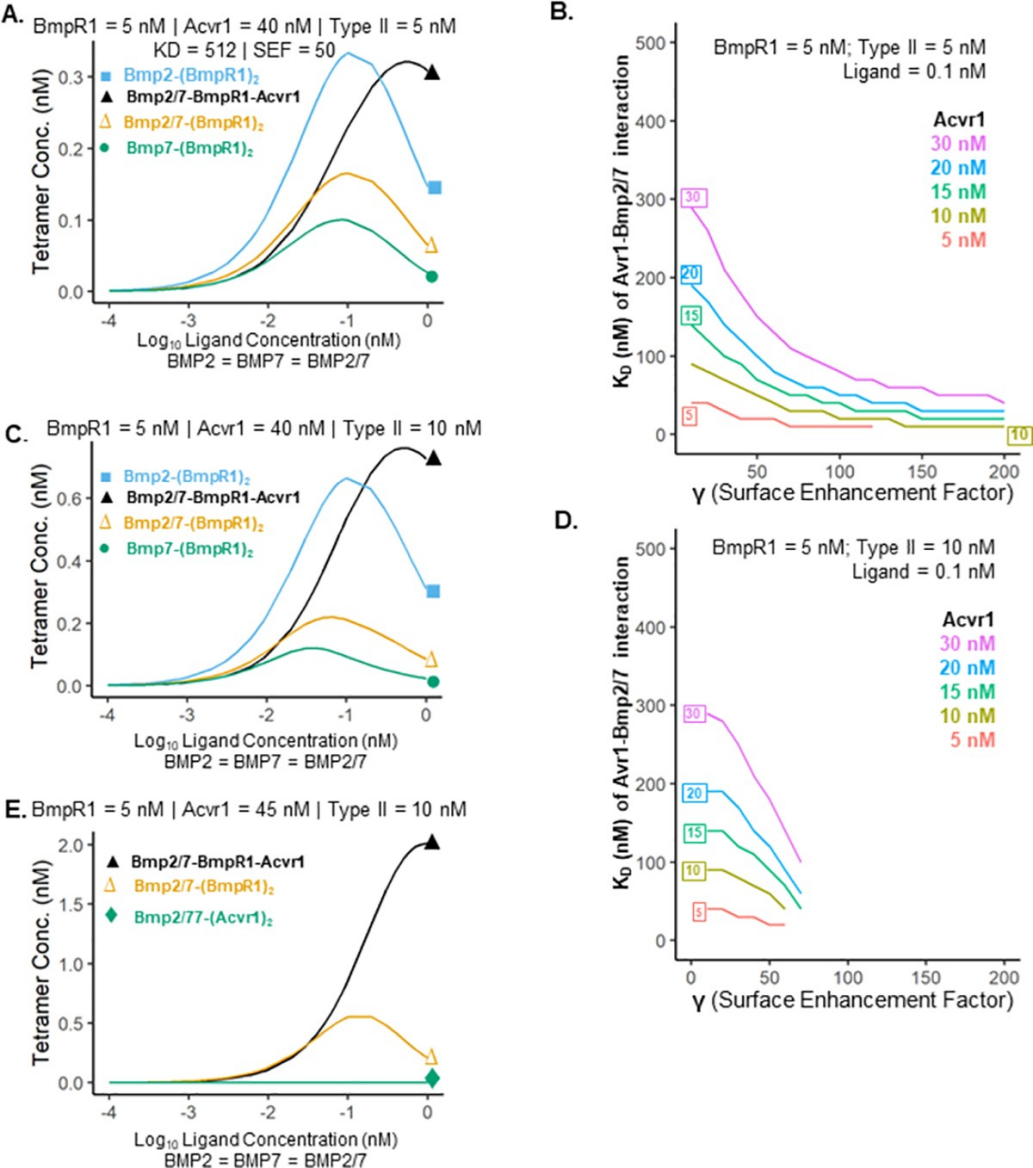
Heterodimer-heterotetramer prevalence under different conditions. **A.** Shows the distributions of the complexes as a function of ligand concentration in a system with excess Acvr1. The level of BmpR1 receptor complexes exceeds the level of Bmp2/7-Acvr1-BmpR1-(Type II)_2_ except for a narrow band from ~.4nM to 1 nM. **B.** The weakest possible Acvr1 –Bmp2/7 binding affinity in which Bmp2/7-Acvr1-BmpR1-(Type II)2 is the most prevalent receptor tetramer at a ligand concentration of 0.1 nM. Recall that higher K_D_ (nM) indicates weaker binding affinity. **C-D.** Same as **A** and **B** with higher amounts of Type II receptor in the model. **E.** In the Bmp2/7-only system with 10nM type II receptor, 9-fold excess Acvr1 leads to greater heterodimer-heterotetramer complex formation over a wide range of ligand concentration.

Using experimentally supported kinetic parameters and equimolar ligand levels our model does not support the hypothesis that heterodimer-heterotetramer signaling is kinetically favored (Fig 2A). With 8-fold higher levels of Acvr1 relative to BmpR1, the network still favors Bmp2-(BmpR1)_2_-(Type II)_2_ homomeric complex formation, except at high ligand concentrations (0.4 to 1 nM of each ligand), where Bmp2/7-Acvr1-BmpR1-(Type II)_2_ becomes the most prevalent receptor complex (Fig 3A and 3C). However, much of this limited range is non-viable for any putative morphogen gradient as it includes conditions in which increased ligand availability leads to decreased receptor signaling (negative slope on the graph).

Current measurements support a weak binding affinity between Bmp2/7 and Acvr1, with a K_D_ of 512 nM to 1024 nM depending on which subunit of the ligand dimer the Acvr1 receptor is binding to [21]. The very weak binding of Acvr1 to Bmp2/7 is likely the limiting factor in the formation of heterodimer-heterotetramer receptor complexes relative to BmpR1 homomeric receptor complexes. To test the sensitivity of our simulation results to this parameter, we measured heterodimer-heterotetramer assembly at Bmp2/7 to Acvr1 binding affinities ranged from 10 nM to 500 nM, under a variety of receptor concentrations and γ factors. We found that under no physiologically plausible combination of binding affinities, γ factors and receptor concentrations was the heterodimer-heterotetramer the predominant receptor tetrameric complex formed. In Fig 3B and 3D, we show the weakest Acvr1 –Bmp2/7 binding affinity at which the heterodimer heterotetramer becomes the most abundant ligand receptor complex. Notably, high Acvr1 concentrations and lower γ factors allow the heterodimer-heterotetramer to be more prevalent than other ligand-receptor complexes at weaker Bmp2/7-Acvr1 binding affinities (Fig 3B and 3D). At higher Type II receptor concentrations, heterodimer-heterotetramer prevalence is only possible at lower γ factors (Fig 3D). Finally, we observe that if homodimers are quelled *in vivo* by BMP antagonists leaving only Bmp2/7 to bind receptors *and* Acvr1 levels are 9-fold higher than BmpR1, the model favors formation of the Bmp2/7-Acvr1-BmpR1-(Type II)_2_ complex over a wide range of ligand concentrations (Fig 3E). This is in contrast to our observation of substantially greater Bmp2/7-(BmpR1)_2_ production at equimolar receptor concentrations (Fig 2B).

**Fig 4:**
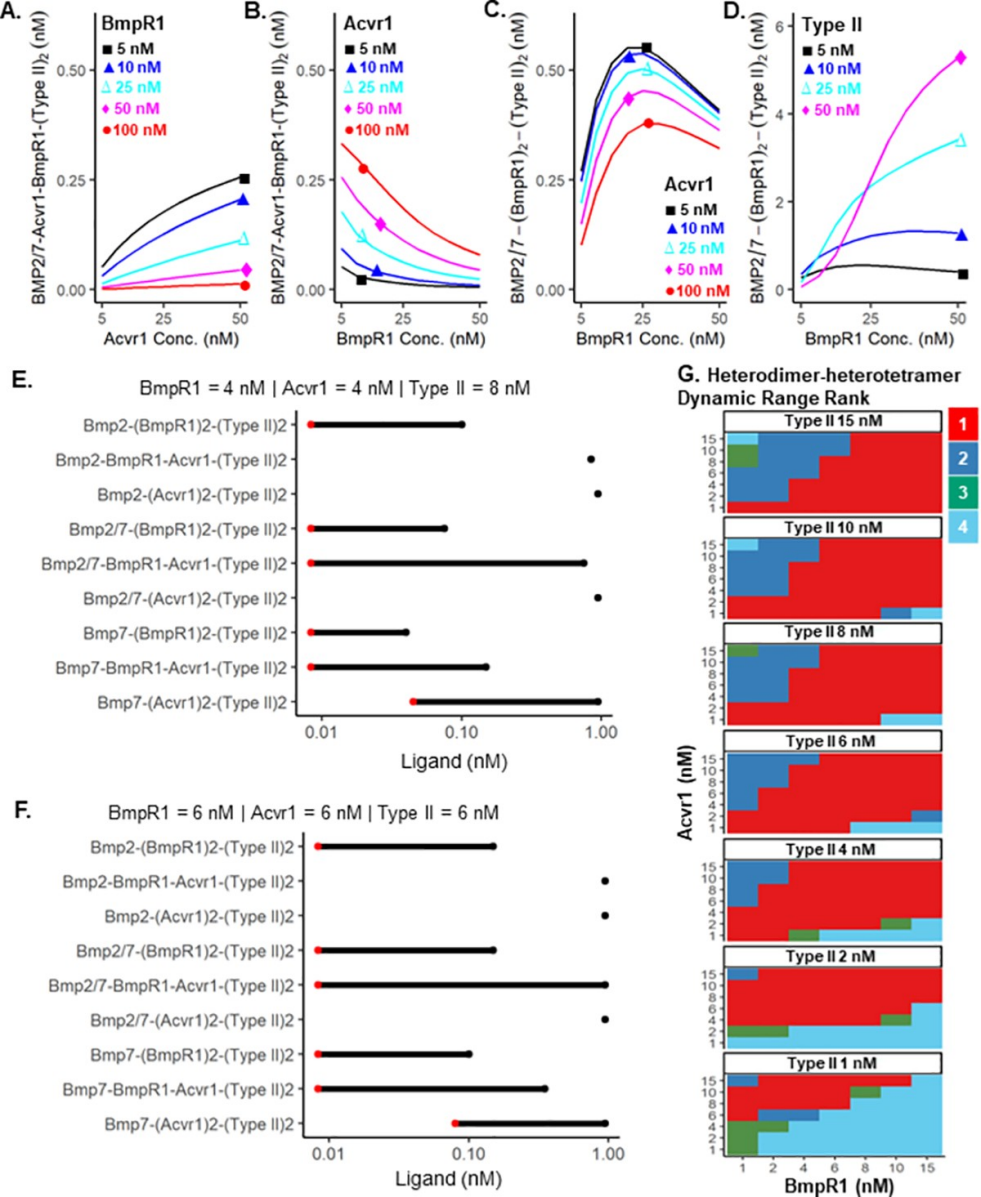
Heterodimer sensitivity and dynamic range. **A-D.** Bmp2 = Bmp7 = Bmp2/7 = 0.3 nM; (A-C) Type II = 10 nM; (D) Acvr1 = 10 nM. (A) Bmp2/7-heteromeric receptor complex formation (y-axis) exhibits low sensitivity to Acvr1 levels (x-axis), but **B.** high sensitivity to increasing BmpR1 levels (x-axis). **C-D.** Levels of BMP2/7-BmpR1-BmpR1 signaling complexes (y-axis) versus increasing levels of BmpR1 for different levels of Acvr1 **C.** or Type II **D.** receptors. **E-F.** Dynamic range, i.e. ligand concentrations needed to produce a single (red point) ligand-receptor complex and maximal ligand receptor complex (black point) for each of the nine plausible tetrameric ligand-receptor oligomers. Oligomers represented by a single point are unable to produce a single tetrameric ligand receptor complex at these ligand and receptor concentrations. The red point signifies, for each tetramer, the higher of a single molecule of ligand, or **G.** Shows a 343 point parameter screen measuring dynamic range under different receptor concentrations. The heterodimer-heterotetramer has the largest dynamic range in 63.56% of the tested conditions.

Recent work suggests that differential kinase activity among BMP receptors is an alternative hypothesis for the primacy of heterodimer signaling [15]. For example, an assumption of high AcvR1 kinase activity and low or zero BmpR1 activity would suggest that ligand-receptor complexes with two BmpR1 receptors, such as Bmp2/2-(BmpR1)_2_-(Type II)_2_ and Bmp7/7-(BmpR1)_2_-(Type II)_2_ have minimal signaling ability. Analysis of our prevalence screen (Fig 2C) under this scenario revealed that after discounting BmpR1 homomeric receptor complexes, the heterodimer-heterotetramer Bmp2/7-Acvr1-BmpR1-(Type II)_2_ is the most abundant signaling complex in a majority (62.39%) of simulations. To further investigate heterodimer-heteromeric receptor signaling in this scenario, we tested the potential contribution of individual receptor levels on expected BMP signaling levels. In model simulations, increasing concentration of Acvr1 leads to increased Bmp2/7 heteromeric receptor signaling at low BmpR1 levels, but has minimal effect at higher BmpR1 concentrations (Fig 4A). Interestingly, an increase in BmpR1 concentration rapidly attenuates Bmp2/7 heteromeric receptor signaling complex formation at both high and low levels of Acvr1 (Fig 4B). In contrast, an increase in BmpR1 concentration leads to a biphasic response in heterodimer-homomeric receptor tetramer (Bmp2/7-(BmpR1)_2_) formation; initially greater BmpR1 levels lead to small to moderate increases in heterodimer-homomeric tetramer complexes, which are reversed as BmpR1 concentrations continue to rise (Fig 4C). This phenomenon appears to be a result of a limiting availability of Type II receptor, as increasing Type II concentration leads to increased heterodimer-homomeric receptor complex formation (Fig 4D). The disparate response to receptor concentrations observed in our model suggests a natural experiment for establishing differential kinase activity among BMP receptors; if Acvr1 indeed has greater kinase activity than BmpR1, than overexpression of AcvR1 should minimally impact observed BMP signaling whereas high levels of overexpression of BmpR1 would lead to a reduction in signaling.

We investigated whether heterodimer-heteromeric receptor signaling confers a performance advantage over homodimer-homomeric receptor signaling for a wide range of ligand concentrations expected in morphogen patterning systems. To be an optimal sensor for a morphogen, the receptors must mitigate noise and respond to a ligand gradient that sets multiple thresholds over space. At two physiologically relevant receptor concentrations, we found that Bmp2-BmpR1 homomeric complexes form over a Bmp2 concentration range of 8.3×10^−3^ nM up to 15×10^−2^ nM where Bmp2 saturates receptors in the model (Fig 4E and 4F). Two aspects suggest that this would be a poor sensor- an extremely low concentration window requires a low number of molecules and potentially increases stochastic noise, and secondly, a relatively narrow band of concentrations between detection and saturation. In the heterodimer model, the heterodimer-heteromeric receptor complexes form over a 6.3 times higher Bmp2/7 concentration range overall, from 8.3×10^−3^ nM up to 0.95 nM (Fig 4E and 4F). We then tested the dynamic range for each tetrameric receptor complex across a 343 point parameter screen of receptor concentrations (Fig 4G). We found that the heterodimer-heterotetramer has a larger dynamic range than BmpR1 homomeric receptor complexes in 82.5% of the parameter space, and the heterodimer-heterotetramer has the highest dynamic range in 63.56% of the parameter space.

In addition to a greater range of ligand responsiveness, these data also show how a heterodimer ligand overcomes a common problem with a tight binding ligand-receptor complex such as BMP2 binding to BmpR1. Tight binding saturates receptors at low ligand concentrations [5,22], such that cells in a BMP gradient are saturated after assembly very near the dissociation constant measurements for the interaction, limiting the formation of a spatial activity gradient that directs differential gene expression over space [22,23]. Indeed, at the lower end of the Bmp2 concentration range less than 0.008 nM, the number of free ligands approaches one molecule or less per cell which is unrealistically low. Notably, the heterodimer-heterotetramer had the highest ligand saturation concentration of all tetrameric complexes for all tested receptor concentrations. Overall, by requiring both BmpR1 and Acvr1, the heterodimer offers the best of both ligand types, it can be recruited rapidly by its interaction with BmpR1, but it also allows a greater dynamic range by binding to the lower affinity Acvr1 before signaling.

## Discussion

In this study, we developed a novel mathematical model of the receptor oligomerization process of membrane receptors in BMP signaling. Our model is consistent with experimental evidence of BMP heterodimer dominance in dorsoventral axis of the zebrafish embryo, and is compatible with known BMP ligand and antagonist activity in this system. The model shows that a simple kinetic based explanation is insufficient to explain the primacy of BMP heterodimer-heterotetramer signaling. While the model disproves a kinetically-driven hypothesis, it indicates the possibility of other mechanisms of limited homodimer signaling; Our model is consistent with recently published evidence for receptor subfunctionalization between the BmpR1 and Acvr1 receptors [15]. Additionally, we demonstrate that heterodimer-heterotetramer complex assembly has properties that are advantageous in morphogen signaling systems. The heterodimer-heterotetramer saturates at higher ligand concentrations than other tetrameric receptor complexes at every tested receptor concentration. Further, at most receptor concentrations, the heterodimer-heterotetramer has the highest dynamic range for ligand interpretation of any receptor tetramer complex. The ability to interpret morphogen levels at higher concentrations suggests that heterodimer-heterotetramer signaling may be more resistant to stochastic noise. Stochastic modeling efforts to understand the noise dynamics of heterodimer signaling are an intriguing area of future study.

BMPs are important growth factors that control various developmental processes both in invertebrates and vertebrates including humans. Evidence shows that disruption of BMP signaling can cause developmental disorders and other diseases. Also, BMPs are very important bio-pharmaceuticals in the treatment of skeletal conditions and in applications of tissue engineering. Studies conducted in this research reveal details of BMP signaling mechanisms, which can be used to develop new bio-pharmaceuticals and treatments of BMP related disorders and diseases. Finally, the computational model developed for ligand dimer-receptor interactions and subsequent oligomerization can be employed to other systems where signaling requires multimerization of membrane receptors, such as the multimerization during the activation of epidermal growth factor (EGF) receptor [24].

To summarize, our *in silico* analysis revealed that the heteromeric complex, Bmp2/7- Acvr1-BmpR1-(Type II)_2_ is the fourth-most prevalent based on the published kinetic rates. Instead, BmpR1 homomeric complexes Bmp2-(BmpR1)_2_-(Type II)_2_, Bmp2/7-(BmpR1)_2_-(Type II)_2_, and Bmp7-(BmpR1)_2_-(Type II)_2_ are more prevalent. This contrasts with the known signal exclusivity or dominance by the heterodimer, suggesting that the increased signaling must be caused by enhanced kinase activity of the heterotetrameric complex. The simulations also provide insight into potential performance advantages afforded by reliance on heterodimer signaling through the heterotetramer receptor complexes, suggesting potential explanations for their presence in morphogen signaling systems.

## Materials and methods

To construct our mathematical model, we devised a biochemical network for BMP ligand-receptor oligomerization that contained all theoretically possible bidirectional interactions between the three ligand-dimers (Bmp2/2, Bmp7/7 and Bmp2/7) and the three BMP receptor species (Type I: BmpR1, Acvr1; Type II). An Ordinary Differential Equation (ODE) was constructed for each of the 51 possible BMP ligand-receptor oligomers; This ODE network simulates each of the 90 possible bidirectional interactions involved in BMP ligand-receptor oligomerization. A schematic of the oligomerization process for each ligand dimer is shown in Fig 1B. Kinetic rates for each of these ODEs are drawn from published biophysical affinity measurements from structural biology studies (S1 Table). Python code for implementing the model is available at https://github.com/akmadamanchi/BMPOligomerizationModel. Our full mathematical model is implemented in python using the PySB library which employs the variable coefficient ode solver (VODE), and facilitates implementation on high-performance computing systems and enables large scale screen of parameters [25,26]. Mathematical equations and full description are included in S2 Text.

The initial interaction between ligand and receptor occurs between a secreted molecule and a surface localized receptor. Subsequent events take place in a smaller, more restricted two-dimensional environment as the remaining receptors needed for tetramer formation are also surface localized. Ligand-receptor interaction rates which are experimentally observed in 3D must be converted to an increased rate of reaction through a general mechanism known as a reduction of dimensionality that has been used to investigate other aspects of BMP-mediated signaling [20,27]. We incorporate the reduction of dimensionality by including the surface enhancement factor (γ) that scales second order binding reactions that occur on a surface by a constant. Typical factors lie between 10 and 100 depending on the cellular structure and the size of the proteins and ligands being patterning and one way to interpret it is that due to the restricted space, the volume for the reaction is much smaller than if it were a well-mixed compartment and all receptors could diffuse around in the entirety of the extracellular space [20,27,28]. The local 2^nd^ order enhancement is specifically due to the spatial restriction and greater concentrating effect of the localization [20].

To adapt experimental biophysical data for the rectangular geometry of the cell membrane and immediate extracellular region we make a reduction of dimensionality adjustment for the secondary and tertiary receptor recruitment processes that take after the initial ligand binding reaction as previously done by us and others [27,29]. Additionally, we make assumptions about the extracellular diffusion of extracellular ligand dimers and the diffusion and transport of membrane-bound receptor complexes that are detailed in S2 Text. This model development process was previously used to model *Dpp* oligomerization in *Drosophila* [28].

### Heterodimer kinetics

The mathematical model we developed is remarkably well supported by biophysical measurements for ligand-receptor binding [27,30,31], leaving relatively few unknowns: 1) the concentrations of ligands, 2) the kinetic rates of heterodimer-receptor binding, and 3) the concentrations of each receptor type.

Numerous crystal structures of BMP ligands, receptors, and their co-crystals show that BMP homodimers have four receptor binding sites, including two equivalent Type I and two equivalent Type II binding sites [21,31–33]. BMP heterodimers, however, have two different Type I sites, as well as two different Type II sites [1]. The Type I sites on the Bmp2/7 heterodimer are each composed of a Bmp2 and Bmp7 binding site.

BMP homodimer:receptor binding affinity [34] results have revealed three relative binding affinities [21,30–32,35–44]: high, low, and very low (S1 Table). For example, Bmp2 binds BmpR1 with high affinity, as does Bmp7 with type II receptors, with dissociation constants (K_D_) of ~0.8 and 6.4nM, respectively [21,31,40]. By comparison, the affinity of Bmp2 for Type II receptors, and Bmp7 for BmpR1, is 10-fold lower, with K_D_’s of 47 and 56 nM, respectively [31,37,39]. The affinity of Bmp7 for Acvr1 is very low, with a K_D_ of~500 nM [31,32,35]. In addition Bmp2 has no demonstrable affinity for Acvr1 [31,35]. We have assumed that the Bmp2/7 ligand heterodimer binds to receptors at the same rate as each of its two ligand monomers: specifically, the Bmp2/7 ligand binds to one Acvr1 receptor at low 512 nM K_D_ affinity for Bmp7, and no or extremely weak affinity (K_D_>1000 nM) for Bmp2; similarly, we assume that Bmp2/7 binds to one BmpR1 receptor at a high affinity Bmp2 domain, and binds another BmpR1 receptor at a low affinity Bmp7 domain. The high affinity site is consistent with the measured affinity of Bmp2/7 to BmpR1 [10]. Endocytosis and ligand/receptor signaling are not included in our model simulation; prior studies have demonstrated that, in the context of steady-state morphogen signaling, endocytosis is constant and the intracellular levels do not contribute to changes in steady-state receptor levels [23,34]

## Supporting information

S1 Table Detailed table of kinetic rates for receptor ligand interactions. (PDF)

S1 Text Chemical Reactions & ODE Equations. (PDF)

S2 Text Detailed text description for reduction of dimension calculations. (PDF)

S1 Fig Graph of parameter space in which heterodimer-heterotetramer is the most abundant tetramer at different ligand levels. (PDF)

S2 Fig Graph of parameter space in which heterodimer-heterotetramer is the most abundant tetramer at different γ levels. (PDF)

## References

[1] Little SC, Mullins MC. Bone morphogenetic protein heterodimers assemble heteromeric type I receptor complexes to pattern the dorsoventral axis. Nature cell biology. 2009;11(5):637. doi: 10.1038/ncb1870 19377468 PMC2757091

[2] Shimmi O, Umulis D, Othmer H, O’Connor MB. Facilitated transport of a Dpp/Scw heterodimer by Sog/Tsg leads to robust patterning of the Drosophila blastoderm embryo. Cell. 2005;120(6):873–86. doi: 10.1016/j.cell.2005.02.009 15797386 PMC6460932

[3] Kishigami S, Mishina Y. BMP signaling and early embryonic patterning. Cytokine & growth factor reviews. 2005;16(3):265–78. doi: 10.1016/j.cytogfr.2005.04.002 15871922

[4] Zinski J, Tajer B, Mullins MC. TGF-β family signaling in early vertebrate development. Cold Spring Harbor perspectives in biology. 2018;10(6):a033274. doi: 10.1101/cshperspect.a033274 28600394 PMC5983195

[5] Umulis D, O’Connor MB, Blair SS. The extracellular regulation of bone morphogenetic protein signaling. Development. 2009;136(22):3715–28. doi: 10.1242/dev.031534 19855014 PMC2766339

[6] Schmierer B, Tournier AL, Bates PA, Hill CS. Mathematical modeling identifies Smad nucleocytoplasmic shuttling as a dynamic signal-interpreting system. Proceedings of the National Academy of Sciences. 2008;105(18):6608–13. doi: 10.1073/pnas.0710134105 18443295 PMC2373357

[7] Matsuda S, Shimmi O. Directional transport and active retention of Dpp/BMP create wing vein patterns in Drosophila. Developmental biology. 2012;366(2):153–62. doi: 10.1016/j.ydbio.2012.04.009 22542596

[8] Bangi E, Wharton K. Dpp and Gbb exhibit different effective ranges in the establishment of the BMP activity gradient critical for Drosophila wing patterning. Developmental biology. 2006;295(1):178–93. doi: 10.1016/j.ydbio.2006.03.021 16643887

[9] Butler SJ, Dodd J. A role for BMP heterodimers in roof plate-mediated repulsion of commissural axons. Neuron. 2003;38(3):389–401. doi: 10.1016/s0896-6273(03)00254-x 12741987

[10] Valera E, Isaacs MJ, Kawakami Y, Belmonte JCI, Choe S. BMP-2/6 heterodimer is more effective than BMP-2 or BMP-6 homodimers as inductor of differentiation of human embryonic stem cells. PloS one. 2010;5(6):e11167. doi: 10.1371/journal.pone.0011167 20567515 PMC2887366

[11] Morimoto T, Kaito T, Matsuo Y, Sugiura T, Kashii M, Makino T, et al. The bone morphogenetic protein-2/7 heterodimer is a stronger inducer of bone regeneration than the individual homodimers in a rat spinal fusion model. The Spine Journal. 2015;15(6):1379–90. doi: 10.1016/j.spinee.2015.02.034 25733023

[12] Buijs J, Van Der Horst G, Van Den Hoogen C, Cheung H, De Rooij B, Kroon J, et al. The BMP2/7 heterodimer inhibits the human breast cancer stem cell subpopulation and bone metastases formation. Oncogene. 2012;31(17):2164. doi: 10.1038/onc.2011.400 21996751

[13] Schmid B, Furthauer M, Connors SA, Trout J, Thisse B, Thisse C, et al. Equivalent genetic roles for bmp7/snailhouse and bmp2b/swirl in dorsoventral pattern formation. Development. 2000;127(5):957–67. 10662635 10.1242/dev.127.5.957

[14] Wang Y-C, Ferguson EL. Spatial bistability of Dpp–receptor interactions during Drosophila dorsal–ventral patterning. Nature. 2005;434(7030):229. doi: 10.1038/nature03318 15759004

[15] Tajer B, Dutko JA, Little SC, Mullins MC. BMP heterodimers signal via distinct type I receptor class functions. Proceedings of the National Academy of Sciences. 2021;118(15). doi: 10.1073/pnas.2017952118 33827919 PMC8054026

[16] Zinski J, Bu Y, Wang X, Dou W, Umulis D, Mullins MC. Systems biology derived source-sink mechanism of BMP gradient formation. Elife. 2017;6:e22199. doi: 10.7554/eLife.22199 28826472 PMC5590806

[17] Tuazon FB, Wang X, Andrade JL, Umulis D, Mullins MC. Proteolytic Restriction of Chordin Range Underlies BMP Gradient Formation. Cell Reports. 2020;32(7):108039. doi: 10.1016/j.celrep.2020.108039 32814043 PMC7731995

[18] Shimmi O, O’Connor MB. Physical properties of Tld, Sog, Tsg and Dpp protein interactions are predicted to help create a sharp boundary in Bmp signals during dorsoventral patterning of the Drosophila embryo. Development. 2003;130(19):4673–82. doi: 10.1242/dev.00684 12925593

[19] Dyson S, Gurdon JB. The interpretation of position in a morphogen gradient as revealed by occupancy of activin receptors. Cell. 1998;93(4):557–68. doi: 10.1016/s0092-8674(00)81185-x 9604931

[20] Lauffenburger DA, Linderman JJ. Receptors: models for binding, trafficking, and signaling: Oxford University Press on Demand; 1996.

[21] Keller S, Nickel J, Zhang J-L, Sebald W, Mueller TD. Molecular recognition of BMP-2 and BMP receptor IA. Nature structural & molecular biology. 2004;11(5):481. doi: 10.1038/nsmb756 15064755

[22] Kerszberg M, Wolpert L. Mechanisms for positional signalling by morphogen transport: a theoretical study. Journal of theoretical biology. 1998;191(1):103–14. doi: 10.1006/jtbi.1997.0575 9593661

[23] Lander AD, Nie Q, Wan FY. Do morphogen gradients arise by diffusion? Developmental cell. 2002;2(6):785–96. doi: 10.1016/s1534-5807(02)00179-x 12062090

[24] Huang Y, Bharill S, Karandur D, Peterson SM, Marita M, Shi X, et al. Molecular basis for multimerization in the activation of the epidermal growth factor receptor. Elife. 2016;5:e14107. doi: 10.7554/eLife.14107 27017828 PMC4902571

[25] Lopez CF, Muhlich JL, Bachman JA, Sorger PK. Programming biological models in Python using PySB. Molecular systems biology. 2013;9(1). doi: 10.1038/msb.2013.1 23423320 PMC3588907

[26] Brown PN, Byrne GD, Hindmarsh AC. VODE: A variable-coefficient ODE solver. SIAM journal on scientific and statistical computing. 1989;10(5):1038–51.

[27] Umulis DM, Shimmi O, O’Connor MB, Othmer HG. Organism-scale modeling of early Drosophila patterning via bone morphogenetic proteins. Developmental cell. 2010;18(2):260–74. doi: 10.1016/j.devcel.2010.01.006 20159596 PMC2848394

[28] Karim MS, Buzzard GT, Umulis DM. Secreted, receptor-associated bone morphogenetic protein regulators reduce stochastic noise intrinsic to many extracellular morphogen distributions. Journal of The Royal Society Interface. 2011;9(70):1073–83. doi: 10.1098/rsif.2011.0547 22012974 PMC3306646

[29] Antebi YE, Linton JM, Klumpe H, Bintu B, Gong M, Su C, et al. Combinatorial signal perception in the BMP pathway. Cell. 2017;170(6):1184–96. e24. doi: 10.1016/j.cell.2017.08.015 28886385 PMC5612783

[30] Kirsch T, Nickel J, Sebald W. BMP-2 antagonists emerge from alterations in the low-affinity binding epitope for receptor BMPR-II. The EMBO journal. 2000;19(13):3314–24. doi: 10.1093/emboj/19.13.3314 10880444 PMC313944

[31] Heinecke K, Seher A, Schmitz W, Mueller TD, Sebald W, Nickel J. Receptor oligomerization and beyond: a case study in bone morphogenetic proteins. BMC biology. 2009;7(1):59. doi: 10.1186/1741-7007-7-59 19735544 PMC2749821

[32] Greenwald J, Groppe J, Gray P, Wiater E, Kwiatkowski W, Vale W, et al. The BMP7/ActRII extracellular domain complex provides new insights into the cooperative nature of receptor assembly. Molecular cell. 2003;11(3):605–17. doi: 10.1016/s1097-2765(03)00094-7 12667445

[33] Harth S. Molecular recognition in BMP ligand-receptor interactions. 2010.

[34] Umulis DM, Serpe M, O’Connor MB, Othmer HG. Robust, bistable patterning of the dorsal surface of the Drosophila embryo. Proceedings of the National Academy of Sciences. 2006;103(31):11613–8. doi: 10.1073/pnas.0510398103 16864795 PMC1544218

[35] Saremba S, Nickel J, Seher A, Kotzsch A, Sebald W, Mueller TD. Type I receptor binding of bone morphogenetic protein 6 is dependent on N-glycosylation of the ligand. The FEBS journal. 2008;275(1):172–83. doi: 10.1111/j.1742-4658.2007.06187.x 18070108

[36] Allendorph GP, Isaacs MJ, Kawakami Y, Izpisua Belmonte JC, Choe S. BMP-3 and BMP-6 structures illuminate the nature of binding specificity with receptors. Biochemistry. 2007;46(43):12238–47. doi: 10.1021/bi700907k 17924656

[37] Allendorph GP, Vale WW, Choe S. Structure of the ternary signaling complex of a TGF-β superfamily member. Proceedings of the National Academy of Sciences. 2006;103(20):7643–8. doi: 10.1073/pnas.0602558103 16672363 PMC1456805

[38] Harth S, Kotzsch A, Hu J, Sebald W, Mueller TD. A selection fit mechanism in BMP receptor IA as a possible source for BMP ligand-receptor promiscuity. PloS one. 2010;5(9):e13049. doi: 10.1371/journal.pone.0013049 20927405 PMC2946932

[39] Isaacs MJ, Kawakami Y, Allendorph GP, Yoon B-H, Belmonte JCI, Choe S. Bone morphogenetic protein-2 and-6 heterodimer illustrates the nature of ligand-receptor assembly. Molecular endocrinology. 2010;24(7):1469–77. doi: 10.1210/me.2009-0496 20484413 PMC2903903

[40] Kotzsch A, Nickel J, Seher A, Heinecke K, van Geersdaele L, Herrmann T, et al. Structure analysis of bone morphogenetic protein-2 type I receptor complexes reveals a mechanism of receptor inactivation in juvenile polyposis syndrome. Journal of Biological Chemistry. 2008;283(9):5876–87. doi: 10.1074/jbc.M706029200 18160401

[41] Kotzsch A, Nickel J, Seher A, Sebald W, Müller TD. Crystal structure analysis reveals a spring-loaded latch as molecular mechanism for GDF-5–type I receptor specificity. The EMBO journal. 2009;28(7):937–47. doi: 10.1038/emboj.2009.37 19229295 PMC2670865

[42] Nickel J, Kotzsch A, Sebald W, Mueller TD. A single residue of GDF-5 defines binding specificity to BMP receptor IB. Journal of molecular biology. 2005;349(5):933–47. doi: 10.1016/j.jmb.2005.04.015 15890363

[43] Weber D, Kotzsch A, Nickel J, Harth S, Seher A, Mueller U, et al. A silent H-bond can be mutationally activated for high-affinity interaction of BMP-2 and activin type IIB receptor. BMC structural biology. 2007;7(1):6. doi: 10.1186/1472-6807-7-6 17295905 PMC1802081

[44] Knaus P, Sebald W. Cooperativity of binding epitopes and receptor chains in the BMP/TGFß superfamily. Biological chemistry. 2001;382(8):1189–95. doi: 10.1515/BC.2001.149 11592400

