# Supplementary material for: Heterodimer-heterotetramer formation mediates enhanced sensor activity in a biophysical model for BMP signaling
Source: PLoS Comput Biol. 2021 Sep 30;17(9):e1009422. doi: 10.1371/journal.pcbi.1009422 (PMC8509922; doi:10.1371/journal.pcbi.1009422)
Supplement: S1 Table — (PDF) [file pcbi.1009422.s001.pdf]

S1 Table:

Receptor	Units	Bmp2		Bmp7		*BMP2/7*		Ref
		Value	K_D (nM)	Value	K_D (nM)	*Value*	*K_D (nM)*	
**1st BmpR1**	k_{on}: $nM^{-1}s^{-1}$<br>k_{off}: s^{-1}	5×10^{-4}<br>4×10^{-4}	0.8	1.4×10^{-4}<br>7.9×10^{-3}	56.4	*5×10^{-4}*<br>*4×10^{-4}*	*0.8*	**1,2**
**2nd BmpR1**	k_{on}: $nM^{-1}s^{-1}$<br>k_{off}: s^{-1}	5×10^{-4}<br>4×10^{-4}	0.8	1.4×10^{-4}<br>7.9×10^{-3}	56.4	*1.4×10^{-4}*<br>*7.9×10^{-3}*	*56.4*	**1**
**1st Acvr1**	k_{on}: $nM^{-1}s^{-1}$<br>k_{off}: s^{-1}	1.2×10^{-6}<br>1.2×10^{-3}	1024	2.3×10^{-6}<br>1.2×10^{-3}	512	*2.3×10^{-6}*<br>*1.2×10^{-3}*	*512*	**1**
**2nd Acvr1**	k_{on}: $nM^{-1}s^{-1}$<br>k_{off}: s^{-1}	1.2×10^{-6}<br>1.2×10^{-3}	1024	2.3×10^{-6}<br>1.2×10^{-3}	512	*1.2×10^{-6}*<br>*1.2×10^{-3}*	*1024*	**1**
**1st Type II**	k_{on}: $nM^{-1}s^{-1}$<br>k_{off}: s^{-1}	1.5×10^{-3}<br>7×10^{-2}	46.6	1.4×10^{-3}<br>9×10^{-3}	6.42	*1.4×10^{-3}*<br>*9×10^{-3}*	*6.42*	**3**
**2nd Type II**	k_{on}: $nM^{-1}s^{-1}$<br>k_{off}: s^{-1}	1.5×10^{-3}<br>7×10^{-2}	46.6	1.4×10^{-3}<br>9×10^{-3}	6.42	*1.5×10^{-3}*<br>*7×10^{-2}*	*46.6*	**3**

Italicized columns indicate that the values for BMP2/7 interaction with BMP receptors are not directly measured, but rather inferred based on homology with BMP2 and BMP7 receptor-binding domains.

1. Heinecke, K. et al. Receptor oligomerization and beyond: a case study in bone morphogenetic proteins. BMC Biol 7, 59 (2009).
2. Saremba, S. et al. Type I receptor binding of bone morphogenetic protein 6 is dependent on Nglycosylation of the ligand. FEBS J 275, 172–183 (2008).
3. Kirsch, T., Nickel, J. & Sebald, W. BMP-2 antagonists emerge from alterations in the low-affinity binding epitope for receptor BMPR-II. EMBO J 19, 3314–3324 (2000).
