# Supplementary material for: Heterodimer-heterotetramer formation mediates enhanced sensor activity in a biophysical model for BMP signaling
Source: PLoS Comput Biol. 2021 Sep 30;17(9):e1009422. doi: 10.1371/journal.pcbi.1009422 (PMC8509922; doi:10.1371/journal.pcbi.1009422)
Supplement: S1 Text — (PDF) [file pcbi.1009422.s002.pdf]

S1 Text: Chemical Reactions & ODE Equations

We show the Chemical Reactions and ODE formulations for BMP2 homodimers only. The complete model can easily be devised by adding similar set of reactions and ODEs for the other two dimers. Additionally, the code for our implementation is provided at github.com/akmadamanchi/BMPOligomerizationModel

Below we show the thirty chemical reactions for the formation of ligand-receptor oligomers involving the BMP2 homodimer. The second receptor unit of each type is designated with the subscript 2

$$BMP2 + BmpR1 \underset{k_{1r}}{\overset{k_{1f}}{\leftrightarrows}} BMP2{:}BmpR1$$

$$BMP2 + Acvr1 \underset{k_{2r}}{\overset{k_{2f}}{\leftrightarrows}} BMP2{:}Acvr1$$

$$BMP2 + TypeII \underset{k_{3r}}{\overset{k_{3f}}{\leftrightarrows}} BMP2{:}TypeII$$

$$BMP2{:}BmpR1 + BmpR1 \underset{k_{4r}}{\overset{k_{4f}}{\leftrightarrows}} BMP2{:}BmpR1_2$$

$$BMP2{:}BmpR1 + Acvr1 \underset{k_{5r}}{\overset{k_{5f}}{\leftrightarrows}} BMP2{:}BmpR1{:}Acvr1$$

$$BMP2{:}BmpR1 + TypeII \underset{k_{6r}}{\overset{k_{6f}}{\leftrightarrows}} BMP2{:}BmpR1{:}TypeII$$

$$BMP2{:}Acvr1 + BmpR1 \underset{k_{7r}}{\overset{k_{7f}}{\leftrightarrows}} BMP2{:}BmpR1{:}Acvr1$$

$$BMP2{:}Acvr1 + Acvr1 \underset{k_{8r}}{\overset{k_{8f}}{\leftrightarrows}} BMP2{:}Acvr1_2$$

$$BMP2{:}Acvr1 + TypeII \underset{k_{9r}}{\overset{k_{9f}}{\leftrightarrows}} BMP2{:}Acvr1{:}TypeII$$

$$BMP2{:}TypeII + BmpR1 \underset{k_{10r}}{\overset{k_{10f}}{\leftrightarrows}} BMP2{:}BmpR1{:}TypeII$$

$$BMP2{:}TypeII + Acvr1 \underset{k_{11r}}{\overset{k_{11f}}{\leftrightarrows}} BMP2{:}Acvr1{:}TypeII$$

$$BMP2{:}TypeII + TypeII \underset{k_{12r}}{\overset{k_{12f}}{\leftrightarrows}} BMP2{:}TypeII_2$$

$$BMP2{:}Bmpr1{:}TypeII + BmpR1 \underset{k_{13r}}{\overset{k_{13f}}{\leftrightarrows}} BMP2{:}BmpR1_2{:}TypeII$$

$$BMP2{:}Acvr1{:}TypeII + BmpR1 \underset{k_{14r}}{\overset{k_{14f}}{\leftrightarrows}} BMP2{:}BmpR1{:}Acvr1{:}TypeII$$

$$BMP2{:}TypeII_2 + BmpR1 \underset{k_{15r}}{\overset{k_{15f}}{\leftrightarrows}} BMP2{:}Bmpr1{:}TypeII_2$$

$$BMP2{:}Bmpr1{:}TypeII + Acvr1 \underset{k_{16r}}{\overset{k_{16f}}{\leftrightarrows}} BMP2{:}BmpR1{:}Acvr1{:}TypeII$$

$$BMP2{:}Acvr1{:}TypeII + Acvr1 \underset{k_{17r}}{\overset{k_{17f}}{\leftrightarrows}} BMP2{:}Acvr1_2{:}TypeII$$

$$BMP2{:}TypeII_2 + Acvr1 \underset{k_{18r}}{\overset{k_{18f}}{\leftrightarrows}} BMP2{:}Acvr{:}TypeII_2$$

$$BMP2{:}BmpR1_2 + TypeII \underset{k_{19r}}{\overset{k_{19f}}{\leftrightarrows}} BMP2{:}BmpR1_2{:}TypeII$$

$$BMP2{:}BmpR1{:}Acvr1 + TypeII \underset{k_{20r}}{\overset{k_{20f}}{\leftrightarrows}} BMP2{:}BmpR1{:}Acvr1{:}TypeII$$

$$BMP2{:}Acvr1_2 + TypeII \underset{k_{21r}}{\overset{k_{21f}}{\leftrightarrows}} BMP2{:}Acvr1_2{:}TypeII$$

$$BMP2{:}BmpR1{:}TypeII + TypeII \underset{k_{22r}}{\overset{k_{22f}}{\leftrightarrows}} BMP2{:}Bmpr1{:}TypeII_2$$

$$BMP2{:}Acvr1{:}TypeII + TypeII \underset{k_{23r}}{\overset{k_{23f}}{\leftrightarrows}} BMP2{:}Acvr1{:}TypeII_2$$

$$BMP2{:}BmpR1{:}TypeII_2 + BmpR1 \underset{k_{24r}}{\overset{k_{24f}}{\leftrightarrows}} BMP2{:}BmpR1_2{:}TypeII_2$$

$$BMP2{:}Acvr1{:}TypeII_2 + BmpR1 \underset{k_{25r}}{\overset{k_{25f}}{\leftrightarrows}} BMP2{:}BmpR1{:}Acvr1{:}TypeII_2$$

$$BMP2{:}BmpR1{:}TypeII_2 + Acvr1 \underset{k_{26r}}{\overset{k_{26f}}{\leftrightarrows}} BMP2{:}BmpR1{:}Acvr1{:}TypeII_2$$

$$BMP2{:}Acvr1{:}TypeII_2 + BmpR1 \underset{k_{27r}}{\overset{k_{27f}}{\leftrightarrows}} BMP2{:}Acvr1_2{:}TypeII_2$$

$$BMP2\colon BmpR1_2\colon TypeII + TypeII \underset{k_{28r}}{\overset{k_{28f}}{\leftrightarrows}} BMP2\colon BmpR1_2\colon TypeII_2$$

$$BMP2\colon BmpR1\colon Acvr1\colon TypeII + TypeII \underset{k_{29r}}{\overset{k_{29f}}{\leftrightarrows}} BMP2\colon BmpR1\colon Acvr1\colon TypeII_2$$

$$BMP2\colon Acvr1_2\colon TypeII + TypeII \underset{k_{30r}}{\overset{k_{30f}}{\leftrightarrows}} BMP2\colon Acvr1_2\colon TypeII_2$$

These 30 chemical reactions are consolidated into 17 ODEs to track the level of each ligand-receptor oligomer. For illustrative purposes the ODE equations for each trimeric oligomer (ligand dimer: Receptor) is included. The γ is applied to each addition reaction after the initial ligand-receptor binding to accommodate for the reduction of dimensionality, or surface enhancement factor. A constant rate of endocytosis is included in each ODE. The full set of ODEs is available in executable code at our github repository linked above.

$$\begin{aligned}\frac{d(BMP2\colon BmpR1)}{dt} &= k_{1f}[Bmp2][BmpR1] - k_{1r}[Bmp2\colon BmpR1] \\ &- \gamma k_{4f}[Bmp2\colon BmpR1][BmpR1] + k_{4r}[Bmp2\colon BmpR1_2] \\ &- \gamma k_{5f}[Bmp2\colon BmpR1][Acvr1] + k_{5r}[Bmp2\colon BmpR1\colon Acvr1] \\ &- \gamma k_{6f}[Bmp2\colon BmpR1][TypeII] + k_{6r}[Bmp2\colon BmpR1\colon TypeII] \\ &- k_{endocytosis}[Bmp2\colon BmpR1]\end{aligned}$$

$$\begin{aligned}\frac{d(BMP2\colon Acvr1)}{dt} &= k_{2f}[Bmp2][Acvr1] - k_{2r}[Bmp2\colon Acvr1] \\ &- \gamma k_{8f}[Bmp2\colon Acvr1][Acvr1] + k_{8r}[Bmp2\colon Acvr1_2] \\ &- \gamma k_{7f}[Bmp2\colon BmpR1][Acvr1] + k_{7r}[Bmp2\colon BmpR1\colon Acvr1] \\ &- \gamma k_{9f}[Bmp2\colon Acvr1][TypeII] + k_{9r}[Bmp2\colon Acvr1\colon TypeII] \\ &- k_{endocytosis}[Bmp2\colon BmpR1\end{aligned}$$

$$\begin{aligned}\frac{d(BMP2\colon TypeII)}{dt} &= k_{3f}[Bmp2][TypeII] - k_{3r}[Bmp2\colon TypeII] \\ &- \gamma k_{10f}[Bmp2\colon TypeII][BmpR1] + k_{10r}[Bmp2\colon BmpR1\colon TypeII] \\ &- \gamma k_{11f}[Bmp2\colon TypeII][Acvr1] + k_{11r}[Bmp2\colon Acvr1\colon TypeII] \\ &- \gamma k_{12f}[Bmp2\colon TypeII][TypeII] + k_{12r}[Bmp2\colon TypeII_2] \\ &- k_{endocytosis}[Bmp2\colon TypeII]\end{aligned}$$
