# Supplementary material for: Heterodimer-heterotetramer formation mediates enhanced sensor activity in a biophysical model for BMP signaling
Source: PLoS Comput Biol. 2021 Sep 30;17(9):e1009422. doi: 10.1371/journal.pcbi.1009422 (PMC8509922; doi:10.1371/journal.pcbi.1009422)
Supplement: S2 Text — (PDF) [file pcbi.1009422.s003.pdf]

S2 Text

A very basic morphogen dispersal mechanism considers a diffusive morphogen with a linear degradation of it, as the morphogen diffuses away upon secretion. Precisely, upon secretion from a source located at $X = 0$, morphogen transports away from the source by a diffusion-decay mechanism. With necessary initial (I.C) and boundary condition (B.C), the morphogen dispersal model is represented as follows:

$$\begin{aligned} \frac{\partial m}{\partial t} &= D_{m0}\frac{\partial^2 m}{\partial x^2} - k_m[m] \\ &B.C: -D_{m0}\frac{\partial m}{\partial x}\Big|_{x=0} = j, \frac{\partial m}{\partial x}\Big|_{x=L} = 0 \\ &I.C: m(x,t=0) = 0 \end{aligned} \tag{1}$$

Units of D_{m0}, k_m is $(length)^2\ \ time^{-1}$ and $time^{-1}$ respectively. The dimensionless form of Eq.1 is obtained by defining two dimensionless variable $\tau = \frac{t}{T}$ and $\xi = \frac{x}{L}$, and the dimensionless form is as follows:

$$\begin{aligned} \frac{1}{T}\frac{\partial m}{\partial \tau} &= \frac{D_{m0}}{L^2}\frac{\partial^2 m}{\partial \xi^2} - k_m[m] \\ &B.C: \frac{\partial m}{\partial \xi}\Big|_{\xi=0} = -\frac{jL}{D_{m0}}, \frac{\partial m}{\partial \xi}\Big|_{\xi=1} = 0 \\ &I.C: m(\xi,\tau=0) = 0. \end{aligned} \tag{2}$$

Rearrangement of Eq.2 gives us three dimensionless quantities, and the terms are: $\frac{1}{Tk_m}$, $\frac{D_{m0}}{k_m L^2}$, and $\frac{jL}{D_{m0}}$. By defining $J = \frac{jL}{D_{m0}}$ and $\lambda^2 = \frac{k_m L^2}{D_{m0}}$, and considering steady state ($\frac{\partial m}{\partial \tau} = 0$) analysis of morphogen dispersal system, we obtain the Sturm-Liouville form of equation with a generalized solution as given below:

$$m_{ss}(\xi) = Ae^{\lambda\xi} + Be^{-\lambda\xi}. \tag{3}$$

Here, A and B are integral constants. The constants A, B can be calculated by taking the spatial derivative of $m_{ss}(\xi)$ with respect to ξ and equating $(\partial m_{ss}/\partial \xi)$ to the two given boundary conditions (B.Cs) as in Eq. 2. From simplification using the B.Cs, the obtained value of the integral coefficients are $A = Be^{-2\lambda}$ and $B = \frac{-J}{\lambda(e^{-2\lambda}-1)}$. By plugging in the values of A and B, and by assuming that λ is large (means that, k_m is large and D_{m0} is small), the steady-state profile of morphogen simplifies to:

$$m(\xi) = \frac{j}{\sqrt{k_m D_{m0}}}e^{-\left(\sqrt{\frac{k_m L^2}{D_{m0}}}\xi\right)}. \tag{4}$$
