# Supplementary material for: Heterodimer-heterotetramer formation mediates enhanced sensor activity in a biophysical model for BMP signaling
Source: PLoS Comput Biol. 2021 Sep 30;17(9):e1009422. doi: 10.1371/journal.pcbi.1009422 (PMC8509922; doi:10.1371/journal.pcbi.1009422)
Supplement: S1 Fig — (PDF) [file pcbi.1009422.s004.pdf]

S1 Fig Graph of parameter space in which heterodimer-heterotetramer is the most abundant tetramer at different ligand levels

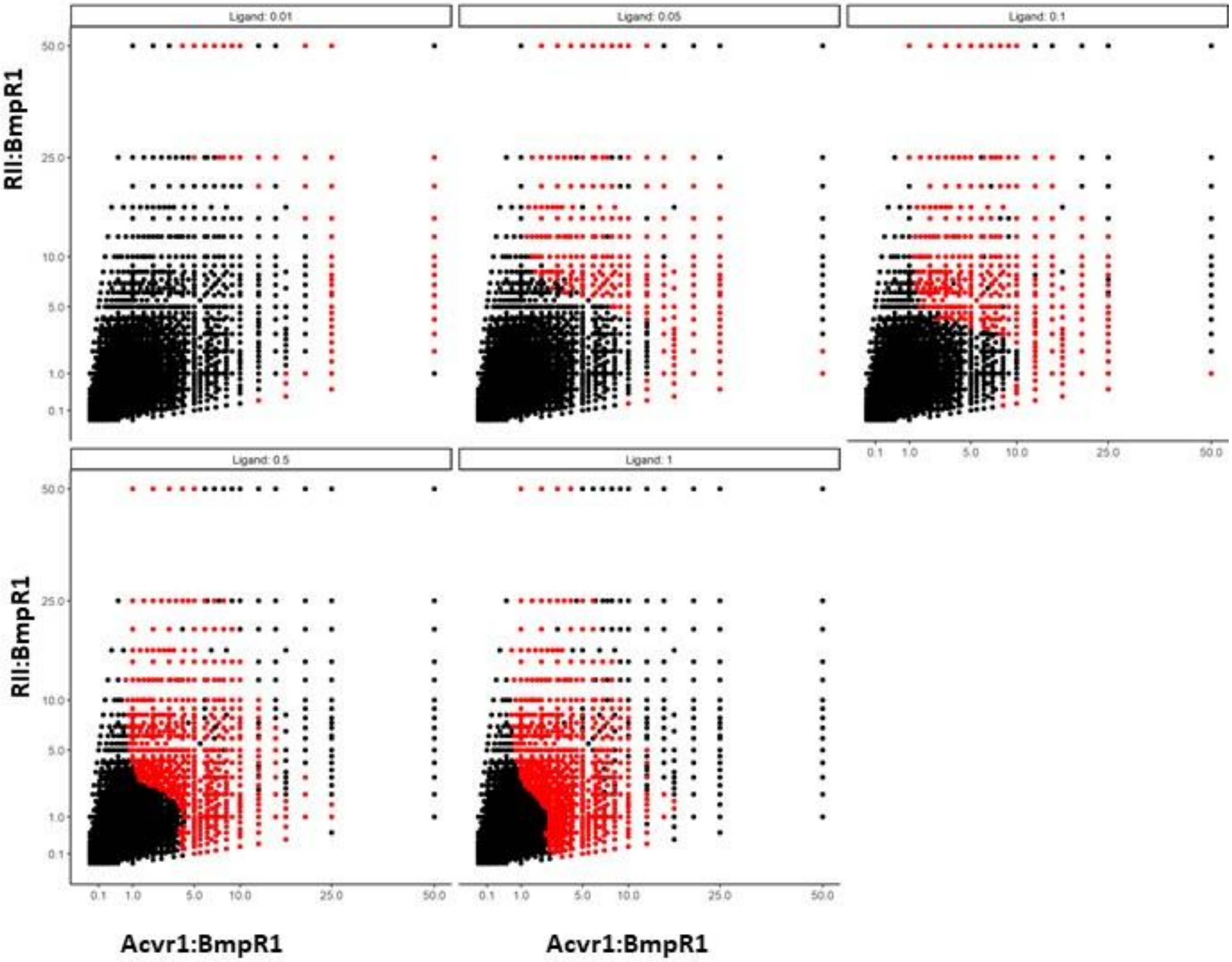

Red shows parameter conditions in which Heterodimer-heterotetrmaer is the most abundant tetramer. Note heterodimer-heterotetramer production is only favored under conditions in which BmpR1 is a limiting factor. There are no conditions in which the heterodimer-heterotramer is preponderant, i.e., makes up a majority of tetramers.
